# Service quality, trust, and patient satisfaction in interpersonal-based medical service encounters

**DOI:** 10.1186/1472-6963-13-22

**Published:** 2013-01-16

**Authors:** Ching-Sheng Chang, Su-Yueh Chen, Yi-Ting Lan

**Affiliations:** 1 R.O.C. Naval academy, Kaohsiung, Taiwan; 2 Division of Nursing, Department of Ophthalmology, Kaohsiung Medical University Hospital, Kaohsiung Medical University, Kaohsiung, Taiwan; 3 Postgraduate Programs in Management, I-Shou University, Kaohsiung, Taiwan

**Keywords:** Interpersonal-based medical service encounters, Service quality, Patient trust, Patient satisfaction, Structural equation modeling (SEM)

## Abstract

**Background:**

Interaction between service provider and customer is the primary core of service businesses of different natures, and the influence of trust on service quality and customer satisfaction could not be ignored in interpersonal-based service encounters. However, lack of existing literature on the correlation between service quality, patient trust, and satisfaction from the prospect of interpersonal-based medical service encounters has created a research gap in previous studies. Therefore, this study attempts to bridge such a gap with an evidence-based practice study.

**Methods:**

We adopted a cross-sectional design using a questionnaire survey of outpatients in seven medical centers of Taiwan. Three hundred and fifty copies of questionnaire were distributed, and 285 valid copies were retrieved, with a valid response rate of 81.43%. The SPSS 14.0 and AMOS 14.0 (structural equation modeling) statistical software packages were used for analysis. Structural equation modeling clarifies the extent of relationships between variables as well as the chain of cause and effect. Restated, SEM results do not merely show empirical relationships between variables when defining the practical situation. For this reason, SEM was used to test the hypotheses.

**Results:**

Perception of interpersonal-based medical service encounters positively influences service quality and patient satisfaction. Perception of service quality among patients positively influences their trust. Perception of trust among patients positively influences their satisfaction.

**Conclusions:**

According to the findings, as interpersonal-based medical service encounters will positively influence service quality and patient satisfaction, and the differences for patients’ perceptions of the professional skill and communication attitude of personnel in interpersonal-based medical service encounters will influence patients’ overall satisfaction in two ways: (A) interpersonal-based medical service encounter directly affects patient satisfaction, which represents a direct effect; and (B) service quality and patient trust are used as intervening variables to affect patient satisfaction, which represents an indirect effect. Due to differences in the scale, resources and costs among medical institutions of different levels, it is a most urgent and concerning issue of how to control customers’ demands and preferences and adopt correct marketing concepts under the circumstances of intense competition in order to satisfy the public and build up a competitive edge for medical institutions.

## Background

The interaction between service provider and customer is the primary core of the service businesses of different natures. The intimate contact between service provider and service recipient is involved in the scenario of service, and such contact opportunity shall definitely and greatly influence the customer’s evaluation process and focal point. One of the important factors is that inseparability exists between production and consumption in such services. Oftentimes, the service encounter system, including service personnel, physical facility, and other tangible elements, is regarded by customers as a part of the service [1]. In addition, the enhancement of service quality places emphasis on the actual service process, and the discussion of service encounter has obviously been the focus of service management [2,3]. Scholars have proposed that trust may allow customer behavior to be more predictable, reduce customer attrition rate, and create higher customer value [4,5]. In addition, trust may further save cost to increase customer satisfaction by creating customer value [6,7], and establish long-term customer relationships [8,9]. Researchers have pointed out that trust imposes positive influence on satisfaction [6,10,11]. Therefore, the influence of trust on service quality and customer satisfaction could not be ignored in interpersonal-based service encounters. However, the lack of existing literature on the correlation between service quality, patient trust, and satisfaction from the prospect of interpersonal-based medical service encounters has created a research gap in previous studies. Therefore, this study attempts to bridge such a gap with an evidence-based practice study.

In the process of service delivery, replacing the simple concept of medical service quality with the concept of interpersonal-based medical service encounters will enhance the dynamic nature and specificity of connotation. Therefore, in recent research, the original service quality studies were replaced by the interpersonal interactive connotation of service encounters [2,9]. However, past studies of the service industries focused mainly on such areas as how to establish long-term seller-buyer relationships, consumer behaviors or customers’ satisfaction with the service quality [1,5]. As for the nature of relationship between the business proprietors and consumers during the process of service delivery, and the phenomenon of different interactive quality between the two, few studies have been conducted, and thus these areas are not sufficiently interpreted. This is particularly true for industries with relatively high level of interaction between the service deliverers and service recipients, such as the ever more prevalent professional service industries in recent years: doctors, lawyers, accountants, etc., in which relatively few empirical studies have been conducted. Nonetheless, it is important that practitioners of the above-mentioned professional services understand customers’ needs during their interaction with the customers, and provide appropriate services to ensure the service quality, so that an edge tool of critical influence can be secured in this highly competitive market today [2]. This is also why the study chooses to explore how the service recipients feel and judge the behavioral performance of professional service providers on the basis of “professional interpersonal-based medical service encounters”. The study aims at seeking to develop and design high-quality medical service solutions, measures, training or public relation activities, etc., in order to improve the medical service quality and the patients’ satisfaction with medical services.

### Hypotheses development and structural framework

#### Relationships among interpersonal-based medical service encounters, service quality, and patient satisfaction

Research has been conducted [12] on service encounters from the viewpoints of customer and service personnel that concluded service personnel and service behavior are both factors for customer’s satisfaction and service quality on service encounter [13]. Therefore, the first-line service provider’s behavior is important in customer’s evaluation of service. Many scholars have suggested that the physical environment where services occur may be helpful to service marketing and it has affected service behavior since earliest trading times, indicating the necessity of proper planning and design of such a physical environment. However, what influence does a service environment have on customers? For general industries, pricing, advertising, and promotion activities are usually more able to attract customers and allow them to feel satisfied with the services more than the physical settings. This phenomenon indicates that physical settings simply play an auxiliary role in terms of the internal corporate goals and external marketing goals for an enterprise. For the service industry, however, things may be different since customers usually arrive and stay at the place where services occur. Hence, strategic planning and space design are apparently more important in the service industry than in other industries.

Booms and Bitner [14] have proposed that the newly added 3Ps in the 7Ps for service industry, physical evidence, participant, and service procedure, might compensate the shortcomings of the traditional 4Ps in marketing: product, place, promotion, and price. Bitner [15] has proposed the “Service Encounter Evaluation Model” to describe the causal variables that influence customer satisfaction or service awareness in the process of service encounter. The 7 Ps of service encountered in consumers’ evaluation of service encounter will influence the contributing factors of the awareness of service performance, service expectation, service quality, and customer satisfaction. From the above statements, the following hypotheses are formed:

##### Hypothesis 1

Perception of interpersonal-based medical service encounters positively influences service quality.

##### Hypothesis 2

Perception of interpersonal-based medical service encounters positively influences patient satisfaction.

#### Relationships among service quality, patient trust, and satisfaction

Rodolfo et al [16] have conducted an empirical study on tourism businesses and explored the functional quality facet and customer trust in service quality. The result suggests that when functional quality of service quality is perceived as superior by consumers, the employees of a service business are more trusted. Foster and Cadogen [17] have confirmed that perceived service quality will significantly and positively influence customer trust. Coulter and Coulter [18] have proposed that service quality is an important preliminary factor to customer trust [19,20]. In their study on service quality and relationship quality, Chang and Chang (2011) [21] and Wong and Sohal (2003) [22] have pointed out that service quality has positive, significant influence on relationship quality (trust, satisfaction, and commitment). Ribbink et al [23] have suggested that service quality has significantly positive influence on trust in their study.

In regard to the relationship between trust and satisfaction [20,24], it has been found that the degree of trust positively influences satisfaction, and furthermore [25,26], that prior trust directly and positively affects consequent satisfaction. Much service-related literature also demonstrates that trust positively influences customer satisfaction. Coulter and Coulter (2003) [19] have indicated that trust is an important factor for the service industry to maintain customer satisfaction. Chiou et al (2002) [27] have proposed in their basic model for customer loyalty that trust will positively influence satisfaction. It has been confirmed in much service-related literature that trust is an important factor in the maintenance of the relationship between service provider and customer satisfaction [13]. Thus, the importance of trust for enterprise and service provider is beyond any doubt. Medical service is a type of intangible product with service encounters; both medical care personnel and general service personnel must develop a trust relationship with patients to enhance patients’ satisfaction. Combining all of the above, the following hypotheses are formed:

##### Hypothesis 3

Perception of service quality among patients positively influences their trust.

##### Hypothesis 4

Perception of trust among patients positively influences their satisfaction.

#### Structural framework of the theoretical relationships

**Figure 1:**
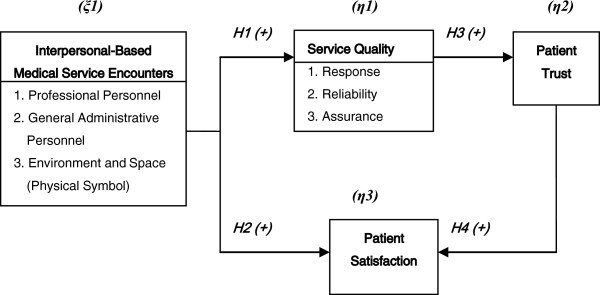
Structural framework of the theoretical relationships.

We thus derive conclusions from the motive, purpose, and article review that the perception in interpersonal-based medical service encounters positively influences service quality and patient satisfaction; the perception of service quality positively influences patient trust; and patient trust positively influences patient satisfaction. The overall research framework is shown as Figure 1.

## Methods

### Research design and participants

In this study, the convenience sampling method was applied to select two medical centers each from northern Taiwan, central Taiwan and southern Taiwan, as well as one from eastern Taiwan, totaling seven medical centers (each was a teaching hospital and medical center, and each had more than 1200 sickbeds). There were a total of eight variables (four constructs) in this study. According to the suggestions provided by Hair et al (1998) [28] regarding sampling in different units of a study, the number of samples should be at least five to ten times the number of research variables in addition to the number of samples for each unit should be more than 30 (that is, a large sample size). Therefore, a unit was then randomly selected from each medical center, where 50 copies of questionnaires were distributed by one trained interviewer of the selected each unit to the outpatients who voluntarily completed the questionnaires. A total of three hundred and fifty questionnaires were distributed between June 2011 and August 2011, and 285 valid ones were collected after incomplete and incorrect questionnaires were filtered out, with a valid response rate of 81.43 %. Israel (2003) [29] has proposed that the more samples drawn, the more statistical significance will be found (but probably the identification of this statistical significance will be meaningless from a management perspective, and it may lead to statistical misrepresentation), thus, they suggested that when the population size is more than 100,000, then, theoretically, the sample size should lie between 204 (95% confidence level and ±7% precision) and 400 (95% confidence level and ± 5% precision). Therefore, the number of questionnaires gathered in this work is consistent with the theoretical sample size.

### Instrument

Because patients directly filled in the questionnaires in the independent variable and dependent variable sections, a single source bias (the deviation caused by the common method variance) might occur [30]. Thus, to avoid and reduce the occurrence of common method variance which might raise the possibility of overestimation and underestimation by the patients, we adopted: 1) a participant information confidentiality approach, using an anonymous method to reassure the participants; 2) a concealed purpose approach, by not revealing the variables of every aspect in the questionnaire to reduce the doubts and suspicions that participants may have. The following is the explanation of the questionnaire, which comprised questions already developed in foreign studies that were modified to serve the study purposes.

First, dimensions of questionnaire forms were obtained from the literature and used to compile questionnaires. Second, the dimensions were slightly modified to create initial questionnaires based on the research purposes and industry features. Third, tests were repeatedly administered to three professors in the industry, and to two medical specialists and five patients; a pilot run of the questionnaire was administered to 37 patients. A total of 32 valid questionnaire forms were gathered, and the results indicated that the reliability was 0.72 to 0.87 (except for one item for the interpersonal-based medical service encounter, two items for patient trust and satisfaction, therefore, these items were deleted) meeting the acceptable standard of more than 0.6 recommended by Chang and Chang (2009) [31]. Finally, the questionnaire was officially released. The questionnaire employed a 5-point Likert scale from 1, for “strongly disagree” to 5, for “strongly agree.” Table 1 summarizes constructs and variables, including operational definitions for all variables. Questionnaires were examined for reliability and validity as follows:

1. Reliability analysis: Principal component factor analysis was used to extract major contributing factors, and varimax of the orthogonal rotation was performed to maximize the differences in factor loading carried by every common factor after the rotation to help recognize common factors. Thus, as Table 1 illustrates, the analytical results indicated that all Cronbach’s α values exceeded 0.6 [28,31,32].

2. Construct convergent validity (confirmatory factor analysis): The confirmatory factor analysis could gain higher recognition than expert content validity [28], and the results for all dimensions are listed in Table 2. All of the adequacy indicators were close to the ideal. Parameters (λ) between each latent variable and manifest variable were estimated to determine the significance of the estimated parameter (λ) in order to evaluate convergent validity. Thus, as Table 2 shows, the t values for the factor loading of all measurement items reached the level of significance (*p< .05*), no single factor included only one question, and the composite reliability values for all constructs were greater than 0.6, which demonstrated satisfactory convergent validity [31,33].

3. Construct discriminant validity: This study performed discriminant validity analysis based on the recommendations of Bagozzi and Yi (1988) and Chang and Chang (2009) [31,33] by limiting the correlation coefficient of the paired dimensions to 1, then performing a Chi-square variance test of the limited and unlimited measurement patterns. If the Chi-square value of the limited pattern exceeds the Chi-square value of the unlimited measurement pattern and reaches a level of significance, then both dimensions have discriminant validity. Thus, as Table 3 shows, the Chi-square values of limited patterns in fact exceeded those of unlimited patterns, and reached a level of significance, indicating discriminant validities among all dimensions.

**Table 1:** Factor naming and reliability analysis results of all the constructs

**Construct/ variable**		**Operational definition**	**Cronbach’s α ( > .6)**	**References**
**Interpersonal-Based Medical Service Encounter**				[1,2]
	Professional Personnel	Patients’ perceptions of the medical professional skill and communication attitude of the physicians and nurses in the service encounter.	**0.92**	
	General Administrative Personnel	Patients’ perceptions of the communication attitude, sympathy, and empathy of the general service personnel in the service encounter.	**0.89**	
	Environment and Space (Physical Symbol)	Patients’ perceptions of the other tangible factors that may help the implementation of medical service in the service encounter.	**0.90**	
**Service Quality**				[34,35]
	Response	Hospital’s capabilities of providing services that instantly and rapidly respond to patients’ demands in the service encounter.	**0.88**	
	Reliability	Hospital’s capabilities of providing services that correctly delivery the service requested by patients in the service encounter.	**0.90**	
	Assurance	Hospital’s capabilities of providing services that really earn patients’ confidence in the service encounter.	**0.93**	
**Patient Trust**				[19,36]
	Patient Trust	Patients’ perceptions of the confidence in the reliability and integrity of medical service in the service encounter.	**0.91**	
**Patient Satisfaction**				[37,38]
	Patient Satisfaction	The psychological state of patient involves their positive or negative feelings or attitudes toward their experience and some specific aspects in the service encounter.	**0.91**	

**Table 2:** Confirmatory factor analysis and convergent validity analysis results of all the constructs

**Construct**	**Variable / question item**	**Standard loading**	**Composite reliability > 0.6**	**AVE > 0.5**
**Interpersonal-Based Medical Service Encounter**	**Professional personnel**		0.85	0.54
	1. I feel the physicians are professional during the whole treatment process.	0.71*		
	2. The physicians will recommend adequate medical treatment according to patients’ needs.	0.71*		
	3. The physicians are able to provide answers that solve my doubts.	0.74*		
	4. The physicians will inform patients about treatment plan.	0.79*		
	5. I feel the nurses are professional during the whole treatment process.	0.72*		
	**General administrative personnel**		0.92	0.74
	1. Service counter personnel are trustworthy.	0.84*		
	2. Service counter personnel are able to take the initiative in assisting the procedures of registration, pharmacy, and cashier to make them smooth and efficient.	0.97*		
	3. Service counter personnel are able to provide answers that solve my doubts.	0.92*		
	4. Service counter personnel have a good service attitude.	0.67*		
	**Environment and space (physical symbol)**		0.91	0.71
	1. The hospital has clear directions inside.	0.85*		
	2. The hospital has wide and comfortable waiting rooms.	0.77*		
	3. The hospital has well-illuminated waiting rooms.	0.90*		
	4. The hospital has clean toilets.	0.84*		
**Goodness of fit: χ**^**2**^**/ *d.f.*= 2.79, GFI= .90, AGFI= .87, NFI= .90, RMSR= .068**[33,39-42]				
**Service Quality**	**Response**		0.77	0.53
	1. The entire service process has a good feedback system and management.	0.56*		
	2. The entire service process allows questions to be answered easily.	0.82*		
	3. The entire service process can complete service in a short period of time.	0.78*		
	**Reliability**		0.94	0.83
	1. The entire service process has complete record of transaction details.	0.94*		
	2. The entire service process is able to correctly complete designated service items.	0.85*		
	3. The entire service process has no error.	0.94*		
	**Assurance**		0.81	0.60
	1. The entire service process can fulfill its promise to customers.	0.75*		
	2. The entire service process has a good security mechanism.	0.97*		
	3. The entire service process is trustworthy.	0.55*		
**Goodness of fit: χ**^**2**^**/ *d.f.*= 2.70, GFI= .96, AGFI= .92, NFI= .96, RMSR= .077**[33,39-42]				
**Patient Trust**	1. Medical care personnel will honestly inform patients about the result of diagnosis.	0.88*	0.87	0.58
	2. Medical care personnel will honor the agreement made with the patients.	0.76*		
	3. My medical issues can be handled through the help from general service personnel.	0.79*		
	4. I can trust medical care personnel’s judgment on my sickness.	0.69*		
	5. I rely on medical care personnel to solve medical issues.	0.66*		
**Goodness of fit: χ**^**2**^**/ *d.f.*= 2.72, GFI= .98, AGFI= .95, NFI= .98, RMSR= .078**[33,39-42]				
**Patient Satisfaction**	1. The entire service provided by the hospital makes me feel happy.	0.89*	0.89	0.68
	2. After consumption, I believe choosing this hospital is a correct decision.	0.85*		
	3. I will recommend the medical service of this hospital to other people.	0.85*		
	4. I am very satisfied with the entire service provided by this hospital.	0.69*		
**Goodness of fit: χ**^**2**^**/ *d.f.*= 2.85, GFI= .99, AGFI= .95, NFI= .99, RMSR= .081**[33,39-42]				

* *p *< .05. AVE= Average variance extracted. χ^**2**^**/ df. = Ratio of Chi-square. GFI = Goodness of Fit Index. AGFI = Adjusted GFI. NFI = Normal Fit Index. RMSR = Root Mean Square of Standardized Residual.**

**Table 3:** Discriminant validity analysis results of all the constructs

**PATTERN**	***χ***^***2***^	***d. f.***	**△*χ***^***2***^
**Interpersonal-Based Medical Service Encounter**			
Unlimited Measurement Pattern	62.35	52	-----
Professional Personnel and General Administrative Personnel	132.08	53	69.73**
Professional Personnel and Environment and Space (Physical Symbol)	116.31	53	53.96**
General Administrative Personnel and Environment and Space (Physical Symbol)	145.22	53	82.87**
**Service Quality**			
Unlimited Measurement Pattern	87.69	31	-----
Response and Reliability	122.58	32	34.89**
Response and Assurance	136.25	32	48.56**
Reliability and Assurance	114.36	32	26.67**

** *p*< .01.

### Data analysis methods

The SPSS 14.0 and AMOS 14.0 (structural equation modeling) statistical software packages were used for data analysis and processing, including:

1. Descriptive statistical analysis: To see the sample characteristics.

1. Structural equation modeling (SEM): According to Scholars [31,43], structural equation modeling clarifies the extent of relationships between variables as well as the chain of cause and effect. Restated, SEM results do not merely show empirical relationships between variables when defining the practical situation. For this reason, SEM was used to test the Hypotheses. This study also used several indices, including Chi-square ratio (< 3), goodness of fit index (GFI> .9), adjusted goodness of fit index (AGFI> .8), normal fit index (NFI> .9) and root mean square of standardized residual (RMSR< .08) to evaluate overall model fitness.

### Ethical approval

Approval for the project was obtained from the Institutional Review Board, the study was then carried out with participants’written consent; each participant’s personal data was kept anonymous and confidential and used only for research purposes to comply with the spirit of the Declaration of Helsinki, 2008. The response period was limited to two months. An introductory letter was attached to the questionnaire to explain the purpose of the study and to ensure respondent confidentiality. Anyone who was also interested in learning about the result of this study was able to request a copy through the contact address provided in the questionnaire.

## Results

### Characteristics of Samples

The respondents included male (50.9%) and female (49.1%) respondents. The demographic data revealed that 30.5% of participants were between 41-50 years of age. Furthermore, most respondents had a senior high school degree or university / college level (36.1% and 31.9% respectively). In terms of occupation, there was considerable variety: service sector (49.5%), commerce (15.1%), industry (11.2%), military, public servant, teacher (10.5%), agriculture (6.0%), freelance (4.2 %), and student (1.1%). Concerning the department in which patients were to receive medical treatment, the internal medicine department and cancer center (38.9% and 20.0% respectively) were visited most frequently by the outpatients (see Table 4).

**Table 4:** Characteristics of samples (N=285)

**Description**	**Frequency**	**Percentage (*%*)**
*Gender*		
Male	145	50.9
Female	140	49.1
*Age*		
20 years and below	19	6.7
21-30 years	26	9.1
31-40 years	68	23.9
41-50 years	87	30.5
51-60 years	56	19.6
61 years and above	29	10.2
*Education level*		
Elementary school and below	27	9.5
Junior high school	53	18.6
Senior high school	103	36.1
University / College	91	31.9
Postgraduate	11	3.9
*Occupation*		
Student	3	1.1
Service sector	141	49.5
Military, public servant, teacher	30	10.5
Industry	32	11.2
Commerce	43	15.1
Agriculture	17	6.0
Freelance	12	4.2
Other	7	2.5
*Department*		
Internal Medicine	111	38.9
Obstetrics & Gynecology	24	8.4
Rehabilitation	39	13.7
Psychiatry (Physical & Mental)	12	4.2
Cancer Center	57	20.0
Ophthalmology	30	10.5
Pediatrics	6	2.1
Other	6	2.1

### Results of structural equation modeling (SEM)

As Table 5 illustrates, Hypotheses in this study are also demonstrated to be statistically significant. Perception of interpersonal-based medical service encounters positively influences service quality (γ_11_= 0.57) and patient satisfaction (γ_31_= 0.23). Perception of service quality among patients positively influences their trust (β_21_= 0.49). Perception of trust among patients positively influences their satisfaction (β_32_= 0.33). Table 5 also shows the results of SEM in this study and the model goodness of fit. In short, it can be concluded that the research model is applicable for the data.

**Table 5:** Results of structural equation modeling

**Path**	**Path name**	**Path coefficient**	***t *Value**
Interpersonal-Based Medical Service Encounter (ξ1)→ Service Quality (η1) **(*H1*)**	γ11	0.57	5.27**
Interpersonal-Based Medical Service Encounter (ξ1)→ Patient Satisfaction (η3) **(*H2*)**	γ31	0.23	2.11*
Service Quality (η1)→ Patient Trust (η2) **(*H3*)**	β21	0.49	4.58**
Patient Trust (η2)→ Patient Satisfaction (η3) **(*H4*)**	β32	0.33	4.26**
Interpersonal-Based Medical Service Encounter (ξ1)→ Professional Personnel (x1)	λ1	0.89	7.21**
Interpersonal-Based Medical Service Encounter (ξ1)→ General Administrative Personnel (x2)	λ2	0.68	5.52**
Interpersonal-Based Medical Service Encounter (ξ1)→ Environment and Space (Physical Symbol) (x3)	λ3	0.77	6.86**
Service Quality (η1)→ Response (y1)	λ4	0.75	6.70**
Service Quality (η1)→ Reliability (y 2)	λ5	0.87	7.03**
Service Quality (η1)→ Assurance (y 3)	λ6	0.97	7.96**
Goodness of fit χ^2^ / *d.f.*= 2.19, GFI = .91, AGFI = .89, NFI = .91, RMSR = .058.			

* *p *< .05. ** *p *< .01.

## Discussion

### Theoretical Implications

The major results in this study are: Perception of interpersonal-based medical service encounters positively influences service quality and patient satisfaction, perception of service quality among patients positively influences their trust, and perception of trust among patients positively influences their satisfaction. The conclusions are discussed, as follows:

Our findings support the statement that perception of interpersonal-based medical service encounters positively influences service quality and patient satisfaction. This agrees with the assertions of previous relevant studies. For example, Booms and Bitner (1981) [14] and Bitner (1990) [15] have respectively suggested that participants play an important role in service quality, including professional personnel, general administrative personnel, and environment and space (physical symbol). As suggested by a previous study by Bitner et al (2000) [44], role expectation and role performance all have an influence on an assessment of service quality and satisfaction by customers after services are delivered and the payment is settled. Also, the role has a significant influence throughout the whole course of a service encounter, and a friendly attitude taken by the service encounter personnel toward customers also has a great influence on service quality and customers satisfaction.

Our findings also support the statement that perception of service quality among patients positively influences their trust and perception of trust among patients positively influences their satisfaction. This agrees with assertions made in previous studies, such as that by Wong and Sohal (2003) [22], which have suggested that service quality is an important antecedent of customer trust. Josep and Velilla (2003) [20] and Singh and Sirdeshmukh (2000) [26] also have indicated that prior trust directly and positively affects consequent satisfaction.

### Practical implications

The results of the study concluded three points to illustrate the importance of the interaction between service provider and patients: 1) the term of role has its meaning in social background. In role theory, any service transaction can be an interactive exchange; 2) the concept of role expectation and the predictability of role may help the understanding of the nature of service transaction; and 3) the element of role in service industry may provide other more useful methods for categorizing service and the marketing method to develop marketing management strategy. Therefore, the results of the study point out the content and structure of the role of professional service provider from the aspects of professional service provider, such as professional personnel as well as the general administrative personnel, and the study also concluded that patients will distinguish between ideal role and expected role and describe professional service role according to the traits of professional ethics, professional skill, communication skull, interpersonal skill and personal characteristic.

Deserving of special attention is that, as interpersonal-based medical service encounter will positively influence service quality and patient satisfaction, and the differences for patients’ perception of the professional skill and communication attitude of personnel in the interpersonal-based medical service encounter will influence patients’ overall satisfaction in two ways: (A) interpersonal-based medical service encounter directly affects patient satisfaction, which represents a direct effect; and (B) service quality and patient trust are used as intervening variables to affect patient satisfaction, which represents an indirect effect. According to the above findings, we learn that patients’ perception of the professional skill and communication attitude of professional personnel in the interpersonal-based medical service encounter also positively influences their satisfaction, with the requirement of service quality and patient trust as the intervening variables. This finding varies from the previous assumption that service quality alone can positively influence patient satisfaction. The results of the study reveal that the higher the patients’ perception of the professional skill and communication attitude of personnel, the better the service quality, patient trust, and patient satisfaction shall be. Based on such findings, the hospitals are recommended to enhance professional skills and communication attitudes of personnel to retain patients and build up a competitive edge for medical institutions as competitive pressure increases.

This study shows that service quality has a positive influence on customer trust, and customer trust also has a positive influence on customer satisfaction. Medical service is a type of intangible product with service encounters; therefore, both medical care personnel and general service personnel must develop a relationship of trust with patients to enhance patient satisfaction. After all, the safety and honesty of the results of diagnosis in the service encounters are now an importance issue due to the openness of the Internet. Customers are anxious about the credibility of the health care provider they deal with, besides illegal interception of their transaction information; therefore, medical institutions must build up customers’ psychological trust in the service encounters. Intangibility of the service industry makes customers unable to predict the result of service; they can only become aware of risk when consumption occurs. Also, consumers of service lack sufficient information and knowledge, and therefore a high level of risk and uncertainty are often inevitable. For the medical service industry, both medical service personnel and general service personnel play an important role in communication, and establishing a relationship of trust with patients is very important.

### Suggestions and recommendations

As the competition in the medical service industry is intense and a large number of medical institutions have to share a limited medical market, medical service marketing (encounter) now plays an important role in hospital management. Also, medical services are still a kind of services, and it is widely recognized that there is inherent difference between medical services and tangible products. Therefore, medical service providers are faced with management challenges about how to overcome specific properties thereof to cater for individual requirements and needs. Since each patient has his/her own demographics, such as cultural background and values, there is significant difference in key factors affecting patients’ choosing medical institutions of different levels as well as decision-making. Owing to the difference in the scale, resources, and costs among medical institutions of different levels, it is the most urgent and concerning issue of how to control customers’ demands and preferences and adopt correct marketing concepts under the circumstances of an intense competition in order to satisfy the public and build up a competitive edge for medical institutions. The following recommendations are made for hospital administrators according to the conclusions of this study:

1. Increasing satisfaction with medical professional personnel will bring the largest rise in overall satisfaction. Nonetheless, “treatment effect” and “doctors’ consultation attitude” are difficult to control, and so top management of hospitals should take doctors’ experience, medical skills and ethics as important criteria when recruiting doctors.

2. Conversely, patients’ perceptions of the communication attitude, sympathy, and empathy of the general service personnel in the service encounter had minimal influence on overall satisfaction, indicating that the general administrative personnel of a hospital have the least effect on service quality, patient trust, and patient satisfaction. The participating hospitals could benefit by carefully interpreting these results.

3. Sustainable operation of a hospital is only possible by keeping a high quality of medical service; understanding patients’ needs and providing customers with the services they expected and needed. From a marketing view, hospital administrators may focus on different customer segments and respond to their needs, in order to increase service quality, and hence the overall patient satisfaction.

4. With the difference in scale, resources, technology, costs, etc., the hospitals may consider their own strengths and weaknesses, and try to upgrade their outpatient clinical service quality and competitiveness by really effective use of their own resources. And hospitals with better facilities and better reputation are usually the first choice of patients and the most important factor.

### Limitations and future studies

Our findings should be considered in view of certain limitations. Firstly, we only investigated the correlation between interpersonal-based medical service encounters, service quality, patient trust, and patient satisfaction in a sample of outpatients. Other factors influencing service quality and patient satisfaction such as the hospital offering convenient transportation, parking, reputable physicians, complexity of disease, and the waiting time, and so on, await further study. Secondly, Cooper and Schindler (2003) [45] and Culyer and Newhouse (2000) [46] have proposed that using aggregation data for inference of individual behaviors might lead to biases. When individual data cannot be observed, using the average value for inference would easily result in biases, because average values cannot reflect individual differences. Therefore, the demographic variables (characteristics of the respondents) were taken as the control variables in this study, but it is expected that individual medical difference can be used as the unit of analysis in future studies to estimate its flexible influence, and hence to conclude the differences in various aspects (constructs) between different hospitals or different specialist departments, etc. Thirdly, the primary research instrument was the questionnaire, which has a certain degree of validity and reliability. However, the results would have been subject to numerous factors that could cause variations in the results, such as defensiveness, misrepresentation, personal emotion and other attitudes. Finally, we examined only one period, which would not reveal factors having long-term effects. A multiple period approach is suggested for follow-up study. Analyzing multiple periods of data would achieve more complete and objective statistical data.

## Conclusions

The 21^st^ century is a new starting point of medical science for Taiwan and other Western countries alike. Since people are giving increasing emphasis to the quality of living, and the demand for medical and health care is growing, Taiwan’s medical care system and the ecology of hospitals have seen changes since the implementation of the National Health Insurance Scheme on March 1, 1995 and the inclusion of some of the medical staff in the applicable scope of the Labor Standards Act: large hospitals have expanded tremendously, with excessive number of beds; this coupling with the general shortage of medical and nursing manpower in hospitals has added enormous workload onto the medical personnel. Hospitalization rate at regional hospitals has dropped significantly, and the number of base-level clinics has increased drastically. All these have resulted in major changes in the internal management and external contingencies of hospitals. The overall operational environment of hospitals is increasingly difficult and full of uncertainties. In response to this drastic change in the operational environment, hospitals are invariably involved in institutional reform and organizational re-engineering by actively strengthening internal quality enhancement, external marketing, forming merger or strategic alliance, etc., in order to boost their own competitiveness. However, escalating costs of medical expenditure and medical insurance have created new problems. Apart from making tighter controls on personnel costs, medical care institutions must also keep their operating costs below a certain percentage while maintaining the level of medical service quality. Hence, in addition to grasping the correct way of development, hospitals should also explore the trend of human resource application in their operation, to make the most out of the manpower at the most economical personnel costs. How to flexibly adjust the number of staff, working hours and manpower structure, making timely rotation of staff, providing sufficient education and training, and making effective distribution and utilization to adapt to the rapid changes in the overall environment and market at any time have become essential issues. This study focuses on “professional interpersonal-based medical service encounters” as the major issue, just in a hope to remind hospitals of the understanding that medical care institutions are a tertiary industry that emphasizes “interaction with people”, that service orientation is demanded, and that patients are of top priority. However, medical care institutions have put too much emphasis on “sales-oriented” rather than “customer-oriented” tasks in the past. In view of the ever-growing patient awareness today, in addition to the current status of supply exceeding demand, medical managers should not only achieve the maximum manpower efficiency and effectiveness with the most streamlined manpower costs, but also adopt a “customer-oriented” operational philosophy, create the “patient value”, work towards the goal of enhancing “patient satisfaction”, and hence actively strive for patient revisits on the basis of “professional interpersonal-based medical service encounters” perspective, so that the ultimate ideal of sustainable operation of the medical care institutions can be achieved.

## Competing interests

The authors declare that they have no competing interests.

## Authors’ contributions

First author CS led the development of this manuscript and contributed to the research design, methodology, and revised draft. Author SY provided the important opinions for the revised draft and secured grant funding. Author YT provided the important opinions for the revised draft and collected the questionnaires. All authors approved and read the final draft.

## Pre-publication history

The pre-publication history for this paper can be accessed here:

http://www.biomedcentral.com/1472-6963/13/22/prepub
